# Favorable and unfavorable health conditions within OECD countries: An exploratory study

**DOI:** 10.1177/2050312117753847

**Published:** 2018-02-13

**Authors:** Myung-Bae Park, Eun Woo Nam, Chun-Bae Kim, Hae Jong Lee, Kwang-Soo Lee, Sang-Baek Koh

**Affiliations:** 1 Department of Gerontal Health & Welfare, Pai Chai University, Daejeon, Republic of Korea; 2 Department of Health Administration, College of Health Science, Yonsei University, Wonju, Republic of Korea; 3 Healthy City Research Center, Institute of Health and Welfare, Yonsei University, Wonju, Republic of Korea; 4 Department of Preventive Medicine, Wonju College of Medicine, Yonsei University, Wonju, Republic of Korea; 5 Institute for Poverty Alleviation and International Development, Yonsei University, Wonju-City, Gangwon-do, Republic of Korea

**Keywords:** Physical health, mental health, social health, health conditions, cluster analysis

## Abstract

**Objectives:**

This study compared the physical, mental, and social health levels among Organization for Economic Co-operation and Development countries.

**Methods:**

We sampled from 34 Organization for Economic Co-operation and Development member countries and divided physical, mental, and social health into three domains based on World Health Organization health definitions.

**Results:**

A multivariate hierarchical cluster analysis was conducted to group countries that were similar in terms of health. Regarding physical health, Japan, South Korea, Sweden, Switzerland, and ten more countries reported favorable health conditions. For mental health, Australia, Canada and eight more countries revealed favorable conditions. Finally, in terms of social health, Austria, Finland, Iceland, and seven more countries reported favorable conditions. Sweden and Switzerland reported the best health conditions aggregated across all three domains. Conversely, Estonia, Hungary, and Turkey reported comparatively poorer health across all three domains when compared with other Organization for Economic Co-operation and Development countries.

**Conclusions:**

We suggested that mental health policy should be further strengthened in cases of Korea and Japan. In case of the Eastern Bloc countries, health policies should be established focusing on health equity for effective improvement of indicators.

## Introduction

The World Health Organization (WHO) defines health as follows: “Health is a state of complete physical, mental and social well-being and not merely the absence of disease or infirmity.”^1^ Generally, physical health is defined as a good, observable physical condition, while mental health refers to a state of well-being wherein individuals recognize their own abilities, can cope with stress, and are capable of making a contribution to their community.^2^ In short, mental health reflects stable emotional conditions, and, thus, is also termed emotional mental health. Social health assumes several meanings such as the ability to form and maintain smooth relationships, have an active social life,^3^ or practice equality and non-violence.^4^

People with good mental and emotional health tend to live longer,^5^ as subjective well-being has a positive effect on physical health.^6,7^ According to previous studies, social health is closely associated with physical health, and social care inequality is known to affect physical health parameters such as morbidity, mortality, and life expectancy.^8^ Furthermore, good social support is associated with positive mental health outcomes.^9,10^ Moreover, socioeconomic situations are related not only to mental but also physical health.^11^ Therefore, we can deduce that physical, mental, and social aspects of health are all associated with one another.

When health levels are compared between countries, life expectancy at birth and mortality rate per 100,000 persons are the most widely used. In fact, the most traditional method is to rank countries according to values on these indices. These indices focus primarily on physical health, as it is difficult to accurately reflect on aspects of mental and social health in the aggregate. For instance, as Japan has the longest life expectancy in the world,^12^ the level of observable physical health could be considered very high; however, the suicide rate in Japan is also very high,^13^ perhaps reflecting lower levels of mental health. In contrast, Turkey and Mexico have the shortest life expectancy but also the lowest suicide rates among all Organization for Economic Co-operation and Development (OECD) countries.^14^ A clear reason for this has not yet been identified, given that socioeconomic and cultural environments differ within each country. Therefore, if one or two health parameters show a positive trend in a particular country, it does not necessarily mean that all aspects of physical, mental, and social health are adequate. Thus, the goal of this study was to compare physical, mental, and social health levels among OECD countries.

## Methods

### Study population and data

A total of 34 OECD member countries were surveyed as of April 2016. We utilized OECD statistics (http://stats.oecd.org/) supplemented with data provided by the WHO and Gallup World Poll. Data from 2012 were used as a baseline, and missing data were replaced with available data for the most recent year. As a result, we used data from 2009 to 2012 for analysis (see Table 2).

### Theoretical model

**Figure 1.**
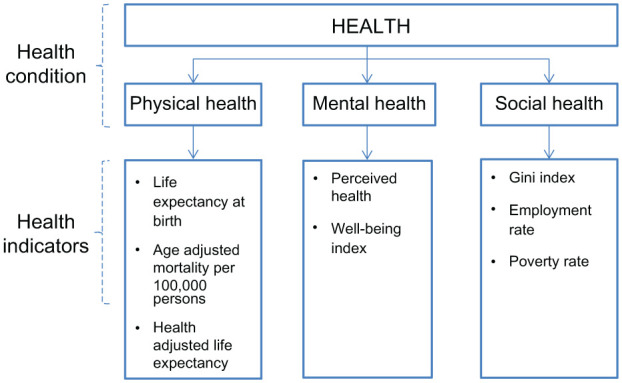
Theoretical model.

To systematically measure health levels, we divided physical, mental, and social health into three domains following WHO definitions. Otherwise, we used eight indicators (Figure 1).

#### Physical health

Population-related health indicators, such as those involving the country, mortality, and morbidity, were used. With respect to morbidity, there were different types, and the deviation was very large due to environmental differences between countries. Because of these features, country classifications were unstable, depending on the variable type included in the analysis. Moreover, morbidity has rarely been used as an analytical index. The age-adjusted mortality rate is generally accepted as a measure of population health. Accordingly, we chose to use age-standardized deaths per 100,000 persons, life expectancy at birth, and health-adjusted life expectancy (HALE) at birth (Table 1).^15^

**Table 1.** Indicators, definitions, and sources.

Domains	Variables	Definition	Sources
Physical health	Life expectancy at birth	How long, on average, a newborn can expect to live	OECD statistics
	Age-adjusted mortality rate	Age-standardized death rates per 100,000 persons for all causes	OECD statistics
	Health-adjusted life expectancy	Average number of years that a person can expect to live in “full health” by taking into account years lived in less than full health due to disease and/or injury	World Health Organization
Mental health	Perceived health status	Percentage of people reporting their health to be “good” or “very good”	OECD statistics
	Well-being index	0 represents the worst possible life and 10 the best possible life	Gallup World Poll
Social health	Gini index	It ranges between 0 in the case of perfect equality and 1 in the case of perfect inequality	OECD statistics
	Employment rate	Total percentage of working age population who are employed	OECD statistics
	Poverty rate	Poverty rate after taxes and transfers and poverty line 50%	OECD statistics

OECD statistics: https://data.oecd.org/ & http://stats.oecd.org/.

World Health Organization: http://www.who.int/gho/publications/world_health_statistics/2015/en/.

Gallup World Poll: http://www.well-beingindex.com/.

#### Mental health

Previous studies have examined mental health using measurement tools to assess negative states such as depression, anxiety, and neurosis. However, since the late 20th century, measurement tools for positive states, such as subjective well-being, happiness, quality of life, and perceived health, have also been used.^16,17^ In this study, we assessed perceived health and well-being as indicators of mental health. Perceived health status (PHS) was represented by the percentage of the population falling under the category of “good” or “very good” with respect to current perceived health. The maximum score for well-being status (WBI) was 10. The higher the value for either index, the more positive the subjective state (Table 1).

#### Social health

Health activity at an individual level, including human relationships and interactions; indices concerning society without discrimination such as nationalism and racism;^18^ and indices of inequality have been used to examine social health.^19^ In this study, a Gini coefficient (which ranges from 0 to 1, where 0 and 1 represent the complete equality and complete inequality conditions, respectively) was used to measure social health. For example, the richest 30% having 80% of all income would have earned income Gini of at least 0.5. Otherwise, 1% of the world’s population owns 60% of all wealth, implying a Gini of at least 0.59. Additionally, employment rates of the working age population, poverty rates, and the ratio of people (in a given age group) whose income falls below the poverty line were used to measure social health (Table 1).

### Statistical analyses

Correlation analyses were performed to examine the associations between health variables. Additionally, a multivariate agglomerative hierarchical cluster analysis was conducted to group countries that were similar in terms of health. Entities with differences across and within groups can be grouped based on certain similarities in their properties.^20^

The levels of physical, mental, and social well-being are not necessarily consistent and are very different depending on the social or cultural context of each country. In such instances, it is difficult to observe the characteristics of each health such as physical, mental, and social if they are integrated en bloc according to score and rank. To overcome this limitation, a cluster analysis that can bind similar groups may be more useful. This method could be useful in health-related studies. It has been used in many studies, including Bae’s study that suggested intervention by grouping Internet-addicted students,^21^ and other studies which involved comparison of dietary patterns of colorectal cancer risk patient,^22^ investigation of a pathogenesis of the condition, and treatment of the people with irritable bowel syndrome through the classification based on their symptom or behavior.^23^

An agglomerative approach, in which the nearest countries were assigned to groups at each iteration step, was used during the grouping process, and the distance between groups was computed using the Ward method. Variables were standardized to a normalized mean of 0 and standard deviation of 1 prior to any analysis due to discrepant measurement scales. All analyses were conducted using Statistical Analysis Software (SAS) Version 9.4. Furthermore, the SAS PROC CLUSTER statement was used for the cluster analysis.

## Results

### Descriptive statistics

The average life expectancy at birth for the whole sample was 80.2 years. Japan had the highest life expectancy (83.2), followed by Iceland (83.0), Switzerland (82.8), and Spain (82.5). Mexico had the shortest life expectancy (74.4), and it was the only OECD nation with a life expectancy below 75. South Korea’s life expectancy was about 1 year above the OECD average (80.2) at 81.3 years. The average adjusted mortality rate was 813.2 per 100,000 people. Japan had the lowest mortality rate (632.8 per 100,000 people), followed by Australia (673.3 per 100,000 people), Switzerland (678.9 per 100,000 people), France (679.3 per 100,000 people), Spain (697.2 per 100,000 people), and Italy (699.2 per 100,000 people), all of which had respectable rates under 700 per 100,000 people. Conversely, the Slovak Republic (1,188.5 per 100,000 people) and Hungary (1,185.1 per 100,000 people) had the highest Mortality, with rates almost double those of the lowest countries. HALE was highest in Japan (75 years); Australia, Spain, Italy, and South Korea ranked second (73 years). The average HALE was 71 years and was shortest in Turkey and Hungary (65 years). PHS was highest in New Zealand (89.3%), followed by Canada (88.8%) and the United States (87.5%). PHS was lowest in South Korea (33.3%), which was less than half the OECD average (69.3%). On the WBI, Denmark (7.8) had the highest value, followed by Canada (7.7) and Norway (7.6). Hungary (4.7) had the lowest value, followed by Portugal (4.9) and Estonia (5.1). The average score on the Gini index was 0.32. Denmark, Slovenia, Norway, and Iceland had the lowest scores (0.25). Income inequality was most severe in Chile (0.50). The average employment rates was 66%, with Iceland scoring highest (80%), followed by Switzerland (79%), Norway (76%), and the Netherlands (75%). Turkey was the lowest (49%), followed by Greece (51%) and Spain (56%). The average poverty rate was 11.4%. Iceland and the Czech Republic had the lowest poverty rates (5.9%), and Israel (20.9%) and Mexico (20.4%) had the highest (Table 2).

**Table 2.** Health indicators among OECD countries.

Country	Life expectancy at 0 (year)	Age-adjusted mortality rate	HALE (year)	PHS (%)	WBI (0 to 10)	Gini (0 to 1)	Employment rate (%)	Poverty rate (%)
**Mean**	**80.2**	**813.2**	**71**	**69.3**	**6.7**	**0.32**	**66**	**11.4**
**Max**	**83.2**	**1188.5**	**75**	**89.3**	**7.8**	**0.50**	**80**	**20.9**
**Min**	**74.4**	**632.8**	**65**	**30.0**	**4.7**	**0.25**	**49**	**5.9**
Australia	82.1	673.3	73	85.4^a^	7.4	0.32	72	14.4^b^
Austria	81.0	763.1	71	70.0	7.3	0.28^a^	73	9.0^a^
Belgium	80.5	823.0	71	74.3	6.9	0.26^b^	62	9.6b
Canada	81.5	689.8^a^	72	88.8	7.7	0.32^b^	72	11.7^a^
Chile	78.9	823.9	70	59.1c	6.6	0.50^b^	62	17.8^a^
Czech Republic	78.2	1004.4	69	60.4	6.2	0.26^b^	67	5.9^a^
Denmark	80.1	857.9	70	70.8	7.8	0.25^b^	73	6.0^a^
Estonia	76.5	1031.0	67	52.4	5.1	0.32^b^	67	11.7^a^
Finland	80.7	789.1	71	67.1	7.4	0.26	70	7.5^a^
France	82.1	679.3	72	68.1	6.8	0.31^b^	64	8.0^a^
Germany	81.0	786.8	71	65.3	6.7	0.29^b^	73	8.7^a^
Greece	80.7	801.6	71	74.8	5.8	0.34^b^	51	15.2^a^
Hungary	75.2	1185.1	65	57.6	4.7	0.29	57	6.8^c^
Iceland	83.0	749.3	72	76.9	6.9	0.25^b^	80	5.9^a^
Ireland	81.0	775.4	71	83.1	7.3	0.30^b^	59	9.7^a^
Israel	81.8	707.7	72	83.5	7.4	0.38^b^	67	20.9^a^
Italy	82.3	699.2	73	68.4	6.4	0.32^b^	58	12.6^a^
Japan	83.2	632.8	75	30.0^c^	6.1	0.34	71	16.0^c^
South Korea	81.3	753.9	73	33.3	6.1	0.31	64	15.2
Luxembourg	81.5	755.1	72	73.8	7.1	0.28^b^	66	8.1
Mexico	74.4	972.0	67	65.5	6.8	0.48	61	20.4^b^
Netherlands	81.2	768.8	71	75.6	7.5	0.28	75	7.2^b^
New Zealand	81.5	727.2	72	89.3	7.2	0.32	72	9.8^a^
Norway	81.5	762.3	71	78.8	7.6	0.25^b^	76	7.7^a^
Poland	76.9	1020.3	67	57.7	5.8	0.30^b^	60	11.2^a^
Portugal	80.5	795.9	71	48.1	4.9	0.34^b^	62	11.9^a^
Slovak Republic	76.2	1188.5	67	65.6	6.1	0.26^b^	60	8.3^a^
Slovenia	80.2	851.7	69	63.1	6.1	0.25^b^	64	8.9^a^
Spain	82.5	697.2	73	74.3	6.2	0.34^b^	56	15.1^a^
Sweden	81.8	743.5	72	81.1	7.5	0.27^b^	74	9.7^a^
Switzerland	82.8	678.9	72	81.9	7.5	0.29^b^	79	10.3^a^
Turkey	74.6	848.6	65	68.6	5.5	0.41^b^	49	19.2^a^
United Kingdom	81.0	790.6	71	74.7	7.0	0.34^b^	71	9.5^a^
United States	78.7	822.8^a^	69	87.5	7.2	0.39^b^	67	17.1^a^

HALE: health-adjusted life expectancy at birth; PHS: perceived health status; WBI: well-being index.

Data from 2012 were used as a baseline. Mortality rate was presented per 100,000 persons.

a Data from 2011.

b Data from 2010.

c Data from 2009.

### Correlation analyses

There were strong correlations between the physical health indices, specifically life expectancy, mortality, and HALE. These three indices were also significantly related with WBI and employment rates. The mental health indices, PHS and WBI, showed a strong correlation with each other, and WBI had a significant relationship with employment rates, as well as with life expectancy, mortality rate, and HALE. Furthermore, the relationships between the social health indices, Gini, employment rates, and poverty rates were all statistically significant (Table 3).

**Table 3.** Correlations between each variable.

	Life expectancy at birth	Age-adjusted mortality rate	HALE	PHS	WBI	Gini index	Employment rate	Poverty rate
Life expectancy at birth	**1**							
Mortality rate	**−0.859†**	**1**						
HALE	**0.952†**	**−0.868†**	**1**					
PHS	0.235	−0.276	0.111	**1**				
WBI	0.523**	−0.543 **†**	0.486**	**0.666†**	**1**			
Gini index	−0.344	−0.034	−0.177	−0.070	−0.158	**1**		
Employment rate	0.521**	−0.372*	0.441**	0.246	0.652 **†**	−0.398*	**1**	
Poverty rate	−0.191	−0.178	−0.013	−0.095	−0.157	**0.847†**	−0.428**	**1**

HALE: health-adjusted life expectancy at birth; PHS: perceived health status; WBI: well-being index.

* p < 0.05, **p < 0.01, ^†^p < 0.0001.

### Multivariate hierarchical cluster analysis

The number of clusters was determined by considering the point at which the semi-partial R^2^ (SR) value did not show a sharp increase when visualized via a tree procedure model (Supplementary Figures 1). In Figure 1, the SR value approaches 1 as the number of clusters is reduced, which implies a reduction in the homogeneity between clusters. Additionally, the pseudo T^2^ statistic and cubic clustering criterion (CCC) were examined.

Physical health was classified according to life expectancy, mortality rate, and HALE. Group 1 consisted of 13 countries (including Sweden and Switzerland) and showed the best health performance with optimal values for life expectancy, mortality, and HALE (life expectancy = 82.1 years; mortality rate = 706.7 per 100,000 people; HALE = 72.5 years). Group 2 consisted of the 14 countries (including the United Kingdom) that ranked second in terms of physical health (life expectancy = 80.5 years; mortality rate = 800.9 per 100,000 people; HALE = 70.6 years). Group 3 consisted of five countries, including the Czech Republic (life expectancy = 76.1 years; mortality rate = 975.3 per 100,000 people; HALE = 67.0 years), and the Slovak Republic and Hungary were categorized as Group 4 (life expectancy = 75.7 years; mortality rate = 1186.8 per 100,000 people; HALE = 66.0 years).

Mental health was classified according to PHS and WBI. Group 1 comprised 10 countries (including Australia and Canada) with the best mental health (PHS = 83.5%; WBI = 7.0). Group 2 comprised 11 countries (including the United Kingdom) with second-tier mental health (PHS = 69.6%; WBI = 7.0). Eight countries (including Greece and Spain) were assigned to Group 3 (PHS = 66.6%; WBI = 6.0), and the five countries (including South Korea and Japan) with the lowest mental health scores (PHS = 44.3%; WBI = 5.1) were assigned to Group 4.

**Figure 2.**
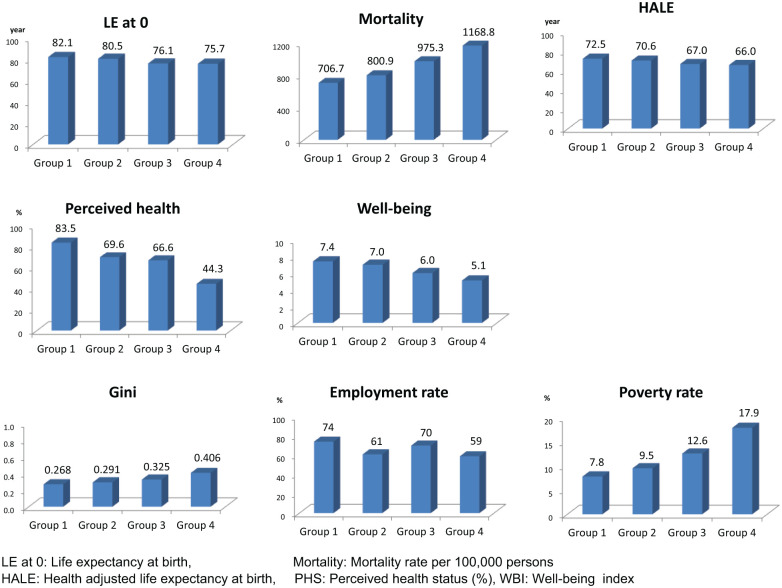
Health status according to physical, mental, and social health groupings.

Social health was classified according to Gini, employment rates, and poverty rates. The 10 countries with the best social health (including Austria, Finland, and Switzerland) were assigned to Group 1 (Gini = 0.268; employment rates = 74%; poverty rates = 7.8%). Group 4, with the worst social health, comprised seven countries, including Chile and Greece (Gini = 0.406; employment rates = 59%; poverty rates = 17.9%). Groups 2 and 3 were intermediate, with Group 2 displaying better Gini and poverty rate scores than Group 3, while Group 3 had a comparative advantage in terms of employment rates. In all, 10 countries (including Ireland and France) were assigned to Group 2 (Gini = 0.291; employment rates = 61%; poverty rates = 9.5%), and 7 countries (including Australia) were assigned to Group 3 (Gini = 0.325; employment rates = 70%; poverty rates = 12.6%) (Table 4; Figure 2).

**Table 4.** Grouping of physical, mental, and social health conditions.

Country	Grouping of physical health	Grouping of mental health	Grouping of social health
Sweden	1	1	1
Switzerland	1	1	1
Iceland	1	2	1
Netherlands	2	1	1
Norway	2	1	1
Australia	1	1	3
Austria	2	2	1
Canada	1	1	3
Denmark	2	2	1
Finland	2	2	1
France	1	2	2
Germany	2	2	1
Ireland	2	1	2
Luxembourg	1	2	2
New Zealand	1	1	3
Belgium	2	2	2
Israel	1	1	4
Italy	1	3	2
Czech Republic	3	3	1
Slovenia	2	3	2
United Kingdom	2	2	3
United States	2	1	4
Chile	2	2	4
Japan	1	4	3
South Korea	1	4	3
Poland	3	3	2
Portugal	2	4	2
Spain	1	3	4
Greece	2	3	4
Mexico	3	2	4
Slovak Republic	4	3	2
Estonia	3	4	3
Hungary	4	4	2
Turkey	3	3	4

Physical and mental health: health levels are highest in Group 1, followed by Groups 2, 3, and 4.

Social health: health level is highest in Group 1, while Groups 2 and 3 are both intermediate, as no group can be deemed better than the other. Health level is lowest in Group 4.

## Discussion

On the whole, countries with good social security, such as Sweden, Switzerland, Iceland, the Netherlands, and Norway, had favorable health conditions. Furthermore, these countries were placed in the top tier in terms of gross domestic product (GDP) and total health expenditures (except for Iceland). Meanwhile, despite having one of the highest GDPs and total health expenditures, the United States was unable to gain a place in the upper group for physical and social health. While the United States managed a place in the top tier in terms of mental health, its placement in Group 2 for physical health could be considered a reflection of its inefficient healthcare system.^24–26^ Moreover, low levels of social health in the United States could be the result of its relative social inequality. The United States is the only OECD nation without a universal insurance system, though it has continually endeavored to improve its healthcare system.^27,28^ From a health sciences perspective, setting the US’s top priority to “reducing and eliminating disparities and inequality” in Healthy People 2020 (in continuation to Healthy People 2010) and attempting to reduce health disparities seems to be appropriate.

Israel was placed in Group 1 in terms of physical and mental health but was included in Group 4 for social health. In the midst of the recent global financial crisis, Israel experienced several hardships. Israel faced severe income disparities, as low-income groups experienced a sharp decline in their earnings (one-fifth their pre-crisis level), whereas the high-income groups increased their earnings.^29^ While this income disparity has gradually decreased recently, Israel’s income inequality continues to be a serious issue compared to other OECD countries. The 2011 Israeli social justice protests are episodic representations of grievances regarding this issue.^30^ Israel’s high social inequality may also be due in large part to their high military expenditures, which limits any social welfare expenditures, leading to a relative shortage of means for counteracting social problems.^31^

Japan had the highest life expectancy and HALE values, and the lowest mortality, within the OECD, but Japan was placed in Group 4 for mental health. Japan’s mortality rate was 20.9 per 100,000 people, with the third highest suicide mortality rate among OECD countries. Meanwhile, the country with the highest suicide mortality rate among OECD countries was South Korea (29.1), with a rate of 2.4 times the OECD average of 12.1.

Despite adequate physical health conditions, mental health in South Korea and Japan seems rather poor. For instance, PHS and WBI levels were extremely low, followed only by Eastern Bloc countries and Turkey. PHS is influenced by various environmental factors. Therefore, it would seem appropriate to develop various social support systems, such as community health programs, that allow individuals to cope well with their environments. South Korea and Japan, with its high suicide mortality rate, should strive to change its social structure and prioritize programs that allow individuals to appropriately cope with any mental health issue.

Estonia, Hungary, the Slovak Republic, and Turkey generally reported poor health conditions. These countries comprised the lower tier for GDP and total health expenditures. These results could stem from these countries’ relatively poor economic conditions and resultant social consequences. Estonia was part of the Soviet Union, sharing a Soviet influence with Hungary and the Slovak Republic. However, unlike these countries, Turkey is not part of the European Union (EU). While Turkey is one of the founding OECD members, it was under strong government-led industrial policies before 1990.

National health levels are positively related with economic power^32,33^ because resource abundance influences health determinants such as nutrition, lifestyle, medical services, and the environment. OECD nations are generally well-developed and have high health levels when compared to the global average.

Joumard et al.^34^ categorized health levels into three groups using data from 2003: life expectancy at birth, life expectancy at 65 years, and infant mortality rate (IMR). Even at the time, Hungary and Turkey were part of the lowest group, and their health levels remained unchanged and below other OECD countries. In a study on 24 OECD countries conducted by the Conference Board of Canada (CBC), Japan had the highest score, with Sweden in second place, and South Korea, Norway, France, and Switzerland jointly assuming the third position.^35^ In this study, Norway, France, and Switzerland had favorable physical, mental, and social health, thus confirming results of the CBC study. However, as South Korea and Japan had the lowest mental health levels and were mid-level in terms of social health, there are some differences between this study and CBC studies. This is mainly due to the CBC ranking index consisting mainly of physical health indices, namely, mortality and morbidity.

When putting together the study results, it is strongly suggested that mental health policy should be further strengthened in cases of Korea and Japan. Also, the overall health index is not good in the Eastern Bloc countries, which is closely related to economic indicators such as GDP and unemployment. Since it is difficult to approach these economic indicators from a health point of view, it is suggested that health policies should be established focusing on health equity for effective improvement of indicators.

This study did not consider grouping overall health levels because a cluster analysis integrates homogeneous factors, and physical, mental, and social health are quite different across different countries. Otherwise, if indicators are homogeneous, they could be tied to rank order clustering. However, if the indicators are heterogeneous, then the factors cannot be ranked from good to poor and will have to be classified according to specific attributes.

Perceived health and well-being are general indicators of mental health, but since they are closely related to physical and social health,^36,37^ they have limitations in terms of difficulty in measuring them as indicators of mental health.^38^ Well-being is an indicator of cultural influence and it is very difficult to compare nations considering their cultural differences. Therefore, subjective indicators can be influenced by socio-cultural factors that make comparison between countries difficult, and therefore, one must be very careful in doing so. Some of the missing indicators have been replaced by their indicators of the last year. Although there was a relatively small variation in numbers, the possibility that it may have affected the grouping cannot be excluded because there was a difference of up to 3 years based on the indicator. Finally, inter-relationships, social capital, and support factors were not considered, as not all countries provided indices for these variables.

## Conclusion

The countries with the best overall health conditions, aggregated across physical, mental, and social indices, were Sweden and Switzerland. These countries were placed in the top tier in terms of GDP and total health expenditures. Estonia, Hungary, and Turkey were comparatively poorer across all three domains when compared to other OECD countries. These countries composed the lower tier for GDP and total health expenditures. Additionally, if even one or two health factors were adequate, this did not necessarily mean that other health factors would be satisfactory. Our results were different from those of previous studies, which primarily focused on physical health. The main strength of this study was the incorporation of mental and social health indicators in determining the health levels among OECD countries. Finally, we suggested that mental health policy should be further strengthened in cases of Korea and Japan. In case of the Eastern Bloc countries, health policies should be established focusing on health equity for effective improvement of indicators.

## Supplementary Material

Supplementary_figure._1 – Supplemental material for Favorable and unfavorable health conditions within OECD countries: An exploratory study Click here for additional data file. Supplemental material, Supplementary_figure._1 for Favorable and unfavorable health conditions within OECD countries: An exploratory study by Myung-Bae Park, Eun Woo Nam, Chun-Bae Kim, Hae Jong Lee, Kwang-Soo Lee and Sang-Baek Koh in SAGE Open Medicine

Supplementary_figure._1_b – Supplemental material for Favorable and unfavorable health conditions within OECD countries: An exploratory study Click here for additional data file. Supplemental material, Supplementary_figure._1_b for Favorable and unfavorable health conditions within OECD countries: An exploratory study by Myung-Bae Park, Eun Woo Nam, Chun-Bae Kim, Hae Jong Lee, Kwang-Soo Lee and Sang-Baek Koh in SAGE Open Medicine

Supplementary_figure._1_c – Supplemental material for Favorable and unfavorable health conditions within OECD countries: An exploratory study Click here for additional data file. Supplemental material, Supplementary_figure._1_c for Favorable and unfavorable health conditions within OECD countries: An exploratory study by Myung-Bae Park, Eun Woo Nam, Chun-Bae Kim, Hae Jong Lee, Kwang-Soo Lee and Sang-Baek Koh in SAGE Open Medicine
