# Supplementary figures and images for: Favorable and unfavorable health conditions within OECD countries: An exploratory study
Source: SAGE Open Med. 2018 Feb 13;6:2050312117753847. doi: 10.1177/2050312117753847 (PMC8826091; doi:10.1177/2050312117753847)

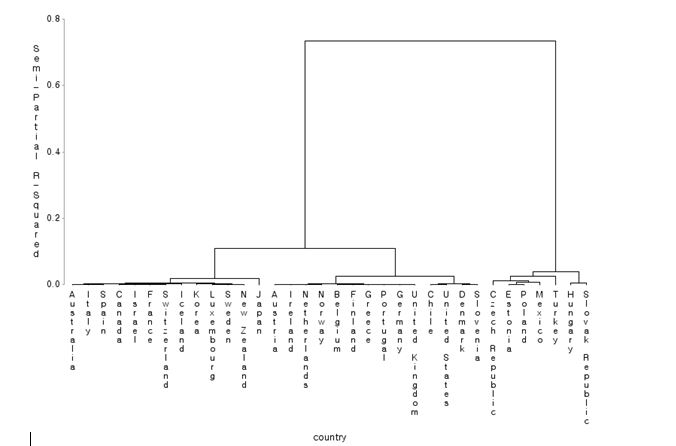

Supplement: Supplementary_figure._1 – Supplemental material for Favorable and unfavorable health conditions within OECD countries: An exploratory study [file Supplementary_figure._1.JPG]

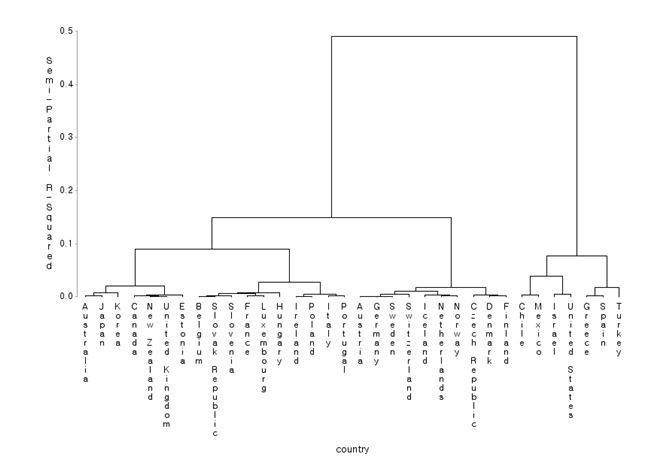

Supplement: Supplementary_figure._1_b – Supplemental material for Favorable and unfavorable health conditions within OECD countries: An exploratory study [file Supplementary_figure._1_b.JPG]

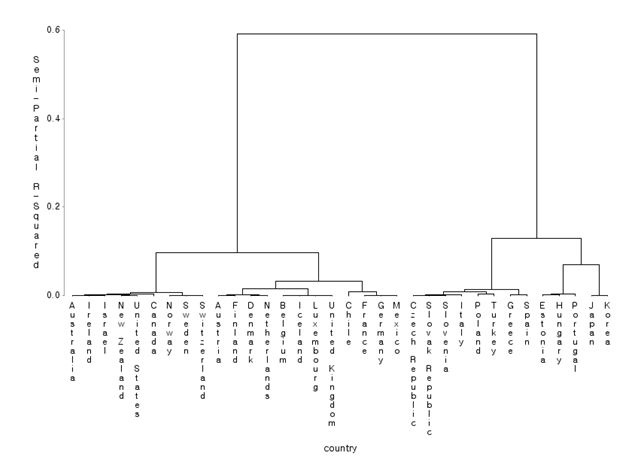

Supplement: Supplementary_figure._1_c – Supplemental material for Favorable and unfavorable health conditions within OECD countries: An exploratory study [file Supplementary_figure._1_c.JPG]
